# Chronic Pelvic Pain, Vulvar Pain Disorders, and Proteomics Profiles: New Discoveries, New Hopes

**DOI:** 10.3390/biomedicines12010001

**Published:** 2023-12-19

**Authors:** Chiara Di Tucci, Ludovico Muzii

**Affiliations:** Department of Obstetrics and Gynecology, “Sapienza” University, 00185 Rome, Italy; ludovico.muzii@uniroma1.it

**Keywords:** chronic pelvic pain, vulvodynia, endometriosis, proteomics, proteomes, protein abundance, proteoforms, biomarkers

## Abstract

Chronic pelvic pain (CPP) is generally defined as non-cyclic pain perceived in the pelvic area that has persisted from three to six months or longer and is unrelated to pregnancy. The etiology of CPP is complex, multifactorial, with heterogeneous presentation, and includes several diseases such as endometriosis, adenomyosis, and interstitial cystitis/bladder pain syndrome. It may also be associated with sexual dysfunction, musculoskeletal disorders, and comorbid psychiatric symptoms. Vulvar pain disorders (VPDs) are typically categorized separately from chronic pelvic pain; among all VPDs, vulvodynia is a chronic vulvar pain of unknown etiology, lasting at least 3 months and that might be associated with other potentially linked factors. Proteomics represents a useful approach to study the proteome profiles of clinical samples. In this review, we have considered a selection of articles that have analyzed the protein abundance and novel protein species from various biological samples, including eutopic/ectopic endometrium, urine, serum, follicular, peritoneal fluid, and cervical mucus, potentially involved in the pathogenesis and progression of CPP and VPDs. These findings could represent valuable targets for paving the way for the differential diagnosis and therapeutic management of CPP and VDPs, thereby optimizing both the prevention and treatment of these conditions.

## 1. Introduction

Chronic pelvic pain (CPP) is generally defined as non-cyclic pain localized in the pelvic area persisting from three to six months or longer and is unrelated to pregnancy. CPP is a condition that affects 25% of the world’s female population, although the cause of the pain is not gynecologic in 80% of the cases [1,2].

The etiology of CPP is complex, multifactorial, with a heterogeneous presentation, and includes several diseases, such as endometriosis, adenomyosis, and interstitial cystitis/bladder pain syndrome. CPP generally refers to pain that is limited to the anatomic pelvis (between the umbilicus and the inguinal ligament). Vulvar pain disorders (VPDs) are typically categorized separately from CPP. Vulvodynia is a VPD of unknown etiology lasting at least 3 months and is usually linked to other potential associated factors or conditions [2,3].

CPP is often related to sexual dysfunction, musculoskeletal disorders, and comorbid psychiatric symptoms. These overlapping symptoms make it extremely difficult to find a consensus on the etiopathogenesis of CPP.

To date, CPP and VPD diagnosis is extremely difficult to obtain; in some cases, the diagnosis arrives about 7–11 years after first appearance of symptoms. At present, many pathological diagnoses can be obtained only by surgery.

Since the early diagnosis of CPP and VPDs is crucial to prevent disease progression, but it is still very difficult to achieve, many efforts are still necessary to identify accurate biomarkers for the diagnosis of CPP without referring patients to invasive or traumatic techniques [4].

A biomarker is a biological indicator that objectively measures health status and the onset or progression of a disease. Biomarkers can identify risk factors or the time when susceptibility is transformed into a disease, and both are gene and protein related [5].

Proteomics is able to identify the structure, function, and abundance of all proteins linked to human physiological mechanisms, the dysregulation of specific molecular pathways, and important differences in signaling pathways [6]. The biological pathways and molecular functions of the target proteins have been implemented in combined studies of bioinformatics and proteomics. Interestingly, all proteins can be studied with analytical hardware, whereas, using available software packages, it has been possible to assemble, select, and analyze proteomics data. In addition, bioinformatics can discover the relationship between molecular regulatory mechanisms and phenotypic behavior, such as disease development and progression [7,8].

Therefore, proteomics technologies can be used to seek new biomarkers for the diagnosis of diseases implicated in CPP and VPDs and to identify novel potential therapeutic targets [6].

Here, we have described the most relevant and recent results obtained from proteomics studies in patients affected by vulvodynia, endometriosis, adenomyosis, and interstitial cystitis, aiming at unveiling novel potential targets for either the diagnosis or treatment of these diseases. Therapeutic tailored interventions to each woman can be obtained with the identification of potential therapeutic biomarkers with the addition of a safety profile.

## 2. Proteomics Methods

Proteomics studies may provide valuable insights about complex processes responsible for the pathogenesis of several diseases [9]. In the context of cancer research, proteomics has helped to identify and characterize protein abundance, the presence of different protein species (proteoforms), and protein interactions, functions, and modifications starting from tissue and biological fluids [10]. Compared to genomic studies, proteomics also characterizes the type and the amount of post translational modifications (PTMs) [9,11], providing a more complex and dynamic picture related to the onset and the development of diseases [10].

The technologies used to evaluate proteomes profiles can be broadly divided into untargeted methods and targeted methods. The first evaluates complex, uncharacterized peptide mixtures from multiple proteins, while the latter focuses on specific proteins or classes of proteins [12]. Targeted methods involve the quantitative analysis of a selection of one or a moderately small number of proteins from a sample [12]. They are usually preferred for more focused studies, providing a more sensitive and absolute quantification of protein concentrations. However, this approach is limited by the predefined sets of labelled canonical peptides offered by companies [9,13].

**Figure 1:**
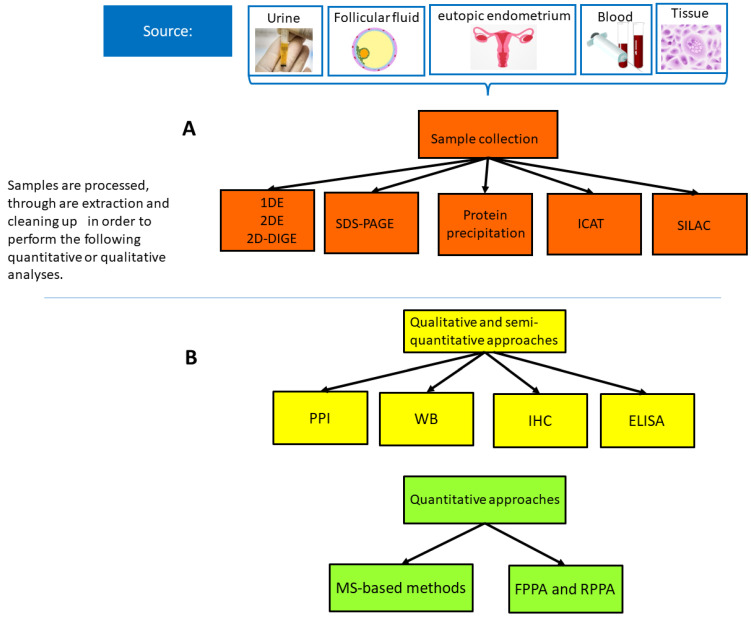
(**A**) Sample collection. (**B**) Qualitative and quantitative approaches. Qualitative, semi-qualitative, and quantitative proteomics strategies used in CPP proteomics studies evaluated. Abbreviations: Protein–Protein Interactions (PPI), Western blot (WT); Immunohistochemistry (IHC); Enzyme-linked immunosorbent assay (ELISA); Mass spectrometry (MS); One- Dimensional Gel Electrophoresis (1DE), Two-Dimensional Gel Electrophoresis, Two-Dimensional Difference Gel Electrophoresis (D-DIGE), stable isotope labelling with amino acids in cell culture (SILAC), isotope-coded affinity tag (ICAT).

Proteomics studies are characterized by a well-controlled multistep process that requires adequate sample preparation, protein separation, protein identification, and final validation steps, followed by statistical and bioinformatics analysis, to acquire accurate, reliable, and reproducible results [10,11]. Conventional methods include chromatography-based techniques (ion exchange chromatography size exclusion chromatography, and affinity chromatography) and enzyme-linked immunosorbent assay (ELISA), which quantify soluble substances in fluids, and Western Blotting (WB) [11]. The latter technique is able to investigate, under a proper control, the presence and relative abundance of PTMs and protein–protein interactions [10]. Co-immunoprecipitation techniques contribute to identify and quantify protein interactomes and PTMs, while immunohistochemistry (IHC), WB, and ELISA can provide quantitative outputs [9,10,11,12,13,14,15] (Figure 1). Results from MS-based studies can be further confirmed using WB and IHC [9,13,16,17,18,19,20,21]. IHC-based methods represent widely used approaches, as they provide information on the biomolecular structure of the organ, tissue cell or even the subcellular level. However, IHC, even in multiplex-based fluorescence microscopy, can detect three to five biomarkers or, with hyperspectral/multispectral methods, up to eight therefore, it remains qualitative or semi-quantitative [13,14] (Figure 1). Immunofluorescence (IF) microscopy is less specific and less reproducible because it targets only one protein species at a time.

Advanced proteomics techniques include protein microarray, liquid chromatography coupled with mass spectrometry (LC-MS/MS), and gel-based approaches, comprising one-dimensional (1DE) and two-dimensional (2DE) gel electrophoresis, 2D difference gel electrophoresis (2D-DIGE), and gel-free high-throughput screening technologies, such as multidimensional protein identification, stable isotope labelling with amino acids in cell culture (SILAC), isotope-coded affinity tag (ICAT), and Isobaric tagging for relative and absolute quantitation (iTRAQ) [10,11]. High-performance LC (HPLC), 1DE, and 2DE are used to separate proteins [11]. 1-DE isolates proteins based on their molecular mass, with a resolving sensitivity ranging from 10 kDa to 300 kDa and is mostly used to characterize proteins after purification [11]. For more complex protein mixtures, such as a crude cell lysate, 2DE may be used; 2DE coupled with WB is able to separate several isoforms of each protein based on their isoelectric point (pI) and molecular weight (MW) [13]. PTM and splicing variants are characterized using MS, particularly tandem MS, whereas protein microarrays might be a useful option for rapid expression analysis, and Edman sequencing allows us to define the amino acid sequence of a specific protein [10,11].

MS is a high-throughput and sensitive technology that allows us to identify the mass of small peptides correlated with specific database values for protein identification. Samples undergo a fractionation step through liquid chromatography before injection into the mass spectrometer (LC-MS), and there can be multiple rounds of MS. MS-based methods are associated with the use of highly specific affinity reagents particularly for proteins with low abundance [9]. Quantification data can be obtained from tissue and cell samples through high-throughput processing, often associated with large-scale WB assays, multiple reaction monitoring assays, label-free quantification of high mass resolution liquid chromatography (LC)-tandem MS [LC-MS/MS], and matrix-assisted laser desorption/ionization (MALDI)-MS/MS-based proteomics [9,11,12,13,14,15,16,17,18,19,20,21,22] (Figure 1). MS-based approaches might also include MALDI-TOF (time-of-flight) MS, SELDI (Surface Enhance Laser Desorption/Ionization )-TOF MS, MALDI-TOF/TOF MS, or MALDI MS [23,24,25,26]. Although MS is the most popular and best analytical technique so far, established approaches like immunoassays still play a crucial methodological role. Immunoassays, protein microarrays, microfluidic-based immunoassays, and other techniques have promising outcomes.

Affinity proteomics methods have emerged as a valuable alternative to MS-based methods, with a detection capability of more than 3000 proteins simultaneously [9,12].

In order to gain comprehensive knowledge, these techniques should complement each other in future studies.

The analysis of proteomics data is a quite complex process due to the extensive amount of information and the scarcity of standards for data formats during processing and data validation steps to create a significant biological association [11]. Bioinformatics is a useful tool to manage the large amount of data and is used in association with other -omics technologies to provide a significant understanding of biological systems and their alterations in human diseases [10,11]. In the present review, we have outlined a brief description of the proteomics technologies applied to CPP and VPD studies. More extensive reviews on proteomics technologies have been published recently that provide additional details [27,28,29].

## 3. Proteomics-Based Investigation of Dysregulated Proteins Involved in Endometriosis, Adenomyosis and Interstitial Cystitis/Bladder Pain Syndrome (IC/PBS), and Vulvodynia

### 3.1. Vulvodynia

Vulvodynia (VVD) is a chronic, multifactorial disease and complex condition with a prevalence ranging from 10% to 28% among reproductive-aged women in the general population [30,31,32]. Diagnosis includes medical history, physical examination, and specific tests but can be reached after the exclusion of bacterial and fungal infections and after differential diagnosis of other causes of pain [33,34].

Almost half of women with VVD might present one of these concomitant conditions: endometriosis (9.8%), fibromyalgia (12.6%), interstitial cystitis, and irritable bowel syndrome (25%).

To date, no specific biomarkers or proteomics abundance data have been identified to study proteins in women with VVD. The lack of knowledge of the differential protein abundance has slowed the development of novel therapies; therefore, current treatment options are diverse and focused on managing symptoms rather than targeting the specific cause of the condition.

MacNeill et al. evaluated protein abundance in eight women with vulvodynia to obtain new potential therapeutic targets [35]. Secretions at vaginal/vestibular sites were collected for protein mapping using gel proteomics, two-dimensional difference gel electrophoresis, and mass spectrometry. Verification of the validity of the results was performed by immunohistochemistry. Annexin A1, interleukin 1 receptor antagonist, protein S100A9, and several antiproteases and proteases were identified in the vestibular fluid of women with vulvodynia The proteins that showed the highest abundance were annexin A1, a protease inhibitor, and the immunoglobulin G κ chain (Table 1). Although proteomics studies on vulvodynia are limited, there are metabolomics and gene expression studies that may help and provide new insights.

Labus et al. studied protein abundance in women with Provoked Vestibulodynia (PVD) [36]. Metabolomics studies on vaginal fluid and plasma in women with PVD have shown that the sphingolipid signaling pathway was expressed at different concentrations in vaginal samples (but not in plasma samples) of women with PVD compared to women without PVD (Table 1). The authors demonstrated that dysregulation in sphingolipid signaling was typically related to local effects on vulvar and vaginal vestibules rather than to systemic effects and involved an increase in vulvar pain and muscle tenderness, sexual dysfunction, and decreased functional connectivity. Alterations in these pathways might represent a new target for therapeutic intervention [36].

### 3.2. Endometriosis

Endometriosis is a common gynecological condition affecting 5–10% of reproductive-aged patients who often present chronic pelvic pain [37]. It is characterized by the presence of endometrial-like tissue outside the uterus which, in most severe cases, can also result in extensive pelvic adhesions and anatomical distortions, leading to pain and infertility.

The diagnosis of endometriosis is obtained by diagnostic laparoscopy, which is the gold standard since it allows for a visual inspection of the pelvic organs. Medical treatment of endometriosis has witnessed major changes in the last years, and progestogens can be considered the first choice in the case of pelvic pain in endometriotic women [38].

Although the pathogenesis of endometriosis is not completely understood, the theory of retrograde menstruation, the tubal backflow of endometrial cells into the pelvis during menses, seems to be the most widely accredited. However, endometriosis occurs with a lower prevalence than menstrual back-flow; therefore, other factors such as genetic predisposition and alterations of immune and endocrine functions should be taken into consideration [39].

In order to gain further insight into the physiopathology of endometriosis and to identify new biomarkers linked to the onset and progression of the disease, several studies have evaluated the role of proteins found in areas potentially involved in proliferation, inflammation, and neo-angiogenesis, including serum, plasma, peritoneal fluid, endometrium, and endometriotic lesions [39,40].

An association between endometriosis and the inflammatory response has been reported. The number of inflammatory cytokines increases in the peritoneal fluid and blood of women affected by this disease compared to healthy women (Table 1). The increase in proinflammatory mediators such as prostaglandins, metalloproteinases, cytokines, and chemokines represents the main mechanism underlining cell metastasis and the invasion of the uterine environment by all immune cells—such as macrophages, natural killer cells, and T cells [41]. Macrophages are involved in endometriosis because of their contribution to adhesion, proliferation, and vascularization, as well as angiogenesis and the innervation processes. Macrophage migration inhibitory factor (MIF) is the most potent factor responsible for endometriosis-related inflammation, as a significant increase in MIF concentrations has been reported in the endometrial tissue of women with endometriosis [42]. A positive correlation was noted between disease stage or the serum estradiol (E2) level and MIF expression. The level of E2-induced MIF upregulation was significantly higher in endometrial cells from women with endometriosis than in cells from women without endometriosis [43] (Table 1).

Bae et al. recently identified differentially expressed genes (DEGs) in three gene expression omnibus microarray datasets in endometriotic tissues [44]. In this study, a total of 135 DEGs were detected in each dataset, mostly belonging to signaling pathways associated with inflammation, complement activation, cell adhesion, and the extracellular matrix. Particularly, the upregulation of seven genes (*C7*, *CFH*, *FZD7*, *LY96*, *PDLIM3*, *PTGIS*, and *WISP2*) out of 17 and the protein expression of four genes (*LY96*, *PDLIM3*, *PTGIS*, and *WISP2*) were identified. The TLR4/NK-kB pathway, as well as estrogen receptors, were involved in the progression of endometriosis (Table 1).

Serum and plasma can be considered the most non-invasive and available biomarkers for endometriosis detection. Sasamoto et al. measured plasma protein levels through a multiplex aptamer- based proteomics platform in adolescents and young adult women with and without endometriosis. In women with endometriosis, 63 proteins were identified and a significant dysregulation in biological pathways related to angiogenesis and cell migration was observed [45] (Table 1).

There is also a direct correlation between endometriosis and the microenvironment of the peritoneal cavity. Four MS-based studies (three 2D-MALDI-MS-TOF and one 2D-LC-MS-TOF) analyzed the proteome profiles of the peritoneal fluid (PF) in women with endometriosis. Ferrero et al., using 2D-PAGE combined with LC-MS/MS, identified a significant higher abundance of eleven proteins in the PF of women with endometriosis when compared to controls [46] (Table 1). They also showed that the amount of protein species (i.e., 1-antitrypsin and S100-A8), frequently upregulated in the PF of women with endometriosis, was significantly higher in subjects with severe disease than in those with mild disease. In addition, the protein species 1- antitrypsin, S100-A8, transferrin, and -1b-glycoprotein had significantly higher abundance in patients with ASRM stage III–IV endometriosis than in patients with ASRM stage I– II disease. However, in women with mild–moderate endometriosis, the most significant protein in terms of abundance was aptoglobin [47] (Table 1).

Ametzazurra et al. studied protein abundance in the endometrial fluid aspirate of women with endometriosis and found that glycodelin was the most frequent protein secreted from the eutopic endometrium of these patients [48] (Table 1).

Another approach to understand the etiology of endometriosis is the study of the eutopic endometrium. There is a differential protein abundance in the eutopic endometrium according to the endometriosis stage, from II to IV [49] (Table 1).

Fowler et al. identified the effect on protein abundance in the eutopic endometrium of humans with endometriosis, focusing on the role of structural and secretory proteins, molecular chaperones, proteins involved in the cellular redox state, and those involved in DNA formation/breakdown [50].

Mear et al. discovered new dysregulated endometrial proteins at any stage of the menstrual cycle, specifically 11 dysregulated biomarkers in the eutopic endometrium [51].

Aromatase P450 is responsible for the conversion of C19 steroids to estrogens in several human tissues (Table 1). Excessive or inappropriate aromatase expression in endometriosis-derived stromal cells is associated with high circulating and local estrogen levels in tissues. A differential aromatase expression amount between the ectopic and eutopic endometrium has been demonstrated in women affected by endometriosis. Noble demonstrated that endometriotic lesions and the eutopic endometrium of women with endometriosis expressed transcripts for P450 aromatase [52]. Interestingly, an increased aromatase expression has been related to the capability of peritoneal surface implantation [53].

Zhang et al. studied protein abundance in the serum and eutopic endometrium of women with stage II, III, and IV endometriosis in the secretory phase and compared it with the serum and eutopic endometrium of a control group [54]. They found a differential expression of 13 proteins from the serum samples that correlated with 11 known proteins and 11 proteins from endometrium samples that correlated with 11 known proteins between women with and without endometriosis. Most of the proteins were components of the cytoskeleton or were involved in the regulation of the cell cycle, signal transduction, or immunological function. Vimentin, beta-actin, and the ATP synthase subunit were differentially expressed between endometriosis sera and normal sera, warranting further investigation in order to elucidate their role in endometriosis pathophysiology.

Ten Have et al. studied the differential biomarker expression of the eutopic endometrium in the secretory phase of the cycle of women affected by endometriosis compared to healthy women [47]. They found 119 proteins differentially expressed between endometriotic and healthy tissues; 21 proteins implicated in the endometriosis sample group were involved in apoptosis, immune reaction, glycolytic pathway, cell structure, and transcription [47].

The application of the 2D-DIGE method identified the presence of species of peroxiredoxin; particularly, one species was specific to the endometrium of women with endometriosis. Analysis of the differentially expressed proteins using the eutopic endometrium has revealed the presence of structural proteins (vimentin, actins), stress response proteins (peroxiredoxins, HSP B1, HSP70, HSP90) or signaling proteins (14-3-3 proteins, annexins), suggesting they might play an important role in the pathology of endometriosis [55,56,57]. Vimentin, a major constituent of the intermediate filament family of proteins, can be found in normal mesenchymal cells and is involved in the control of cellular integrity and plays a role in tumor growth and invasion. Therefore, this protein, in recent years, has been considered a marker for epithelial–mesenchymal transition in endometriosis.

Studies on HSP90 and HSP70 demonstrated that stress proteins were differentially regulated in various stages of endometriosis (Table 1), and the dysregulation of HSPs could be involved in the pathogenesis of endometriosis specifically related to the induction of proliferation and antiapoptotic pathways. Furthermore, annexins, since they are involved in cell proliferation and anti-apoptosis signals, can be involved in the pathology of endometriosis. The differential expression of annexins at various stages of endometriosis suggests a functional role of these proteins in the pathogenesis of this disease [58,59,60].

Endometriosis can be associated with infertility, with a decrease of the number and quality of oocytes, and, therefore, with a negative impact on in vitro fertilization (IVF) outcomes. However, the mechanism by which endometriosis affects oocytes and leads to infertility is not clear. Analysis of the Follicular Fluid (FF) can be considered an important tool for the non-invasive diagnosis of endometriosis and to explore the possible pathogenesis of endometriosis-associated infertility. In an experimental study conducted by Cao et al., elevated GDN (glyco-diosgenin) protein levels were found in the FF of women with endometriosis-associated infertility [61] (Table 1). Combining label-free quantitative proteomics (LFQP) technology and the parallel reaction monitoring (PRM) method, the authors found an abnormal amount of GDN and AGT (glyoxylate aminotransferase) in FF, suggesting that it might be considered a potential cause of endometriosis-associated infertility.

Yao et al. studied the biomarkers involved in the pathophysiology of endometriosis in infertile patients; they compared the overall proteomics profiles of the eutopic endometrium in infertile women with endometriosis with the endometrium of healthy women [62]. A different proteomics abundance was discovered in the eutopic endometrium of the endometriosis group with 16 upregulated proteins and 23 downregulated proteins. Humoral immune response pathways, antimicrobial humoral response, and the regulation of the nitric oxide biosynthetic process were the most involved (Table 1).

### 3.3. Adenomyosis

Adenomyosis can be defined as the infiltration of endometrial glands and stroma into the myometrium and can be related to chronic pelvic pain, dysmenorrhea, dysuria as well as infertility. Multiple genes/signaling pathways can be dysregulated and potentially involved in the development of adenomyosis, but the specific mechanism underlying these processes has not been identified [63,64].

The first study that investigated the role of proteomics as a tool to find potential biomarkers in the pathophysiology of adenomyosis was conducted by Liu et al. [65]. They studied the protein abundance of adenomyosis samples from ten women who underwent hysterectomies and compared them with the protein profiles of normal uterine muscle. By mass spectrometry, they found 12 spots representing dysregulated proteins of the adenomyotic tissue compared to a normal uterus (Table 1).

Xiaoyu et al. studied serum proteins implicated in cell adhesion, the immune response, and the inflammatory response with the iTRAQ (isobaric tags for relative and absolute quantitation) technology [66]. Out of a total of 167 proteins, 25 were abnormally expressed in women with adenomyosis compared to a control group (Table 1).

The same authors discovered a differential protein abundance between endometriosis and adenomyosis samples, mainly because of differences in pathogenesis. Proteins increased in the adenomyosis group were related to blood coagulation and complement activation effects, while those overexpressed in the endometriosis samples were involved in the inflammatory response and the regulation of apoptosis. The upregulation of annexin A2 ANXA2, a member of annexin family proteins, might be related to markers of epithelial-to-mesenchymal transition and is associated with the induction of endometrial cells with both metastatic potential and proangiogenic capacity [67].

Endometrial stromal cells (ESCs) are crucially involved in the pathogenesis of adenomyosis. Liu et al. analyzed protein abundance between eutopic and ectopic ESCs (EuESCs and EcESCs) in adenomyosis samples. LIM and SH3 protein 1 (LASP1), as a cytoskeletal scaffold protein, were expressed in EcESCs compared to EuESCs [50] (Table 1). These results were related to the development of adenomyosis by facilitating the cell proliferation, migration, and invasion of EcESCs, and the authors demonstrated that the dysregulation of LASP1 was implicated in the DNA hypermethylation of the promoter region of the gene.

Extracellular vesicles (EVs) can be derived from every cell type, for example, mast cells, and contain a variety of molecules, such as nucleic acids, cytokines, lipids, and proteins (Table 1). Adenomyosis-derived EVs (AMEVs) may transport specific biomarkers involved in the early diagnosis of adenomyosis. Chen et al., in their recent work, analyzed the expression of tissue or blood AMEVs in women with adenomyosis. HSP90A, STIP1, and TAGLN-2 were overexpressed in these women compared to the control group [68] (Table 1).

### 3.4. Interstitial Cystitis/Bladder Pain Syndrome (IC/PBS)

Interstitial cystitis/bladder pain syndrome (IC/PBS) is a “bladder disease usually characterized by pain, urgency, and frequency, in the absence of urinary tract infection” [69].

There are currently multiple hypotheses explaining the cause of IC/PBS, including bladder epithelial dysfunction, mast cell activation, immune system alterations, and pelvic floor hypertonicity [70,71,72].

Many studies have attempted to find novel protein biomarkers in different biofluids associated with IC/PBS, but none of them were considered worthy to use in the clinical setting. The diagnostic process involves a cystoscopic examination, which is a standard routine for IC/PBS patients and allows the separation of patients into two subtypes, i.e., with and without Hunner’s lesions.

Ward et al. studied the protein abundance in bladder biopsies of both subtypes of IC/PBS patients and evaluated the presence of molecular biomarkers using nanoscale high-performance liquid chromatography tandem mass spectrometry (nHPLC-MS/MS) [73] (Table 1). Women with Hunner’s lesions had a significant increase in the inflammatory setting, with high expression of T- and B-cell markers and endoplasmic reticulum stress proteins compared to the control. A reduction in cellular adhesive proteins and in proteins associated with the Rap1 signaling pathway involved in cell proliferation is the mechanism that induces Hunner’ s lesions (Table 1).

Interestingly, the tissues with Hunner’s lesions showed a decrease in ubiquitination proteins compared to non-disease apparent (NDA) tissues, highlighting the profound dysregulation of cellular adhesion and wound healing signaling. A reduction of urothelial markers, focal lymphoid aggregates in bladder submucosa, and an elevation of immunoglobulin concentration in urine was also described (Table 1). Women with IC/PBS presented high levels of cytokines and chemokines and enhanced immunoreactivity for muscarinic M2, purinergic P2 × 1, P2 × 2, and histamine H1 receptors compared with healthy patients [74,75] (Table 1).

IC/PBS is a bladder-related disease where urine resides for a long time in the bladder, collecting proteins and peptides. Therefore, urine can be considered a fluid biomarker and, through MS analysis, it was possible to discover around 1543 proteins. However, proteomics abundance and protein downregulation in urine were not easy to detect [76].

In women with IC/PBS, urine tyramine and 2-oxoglutarate were significantly elevated compared to the heathy control group (Table 1). Moreover, a high concentration of phenylacetylglutamine was found in the urine of mild-to-moderately affected women [76].

One glycosylated nonapeptide, also known as antiproliferative factor (APF), has been detected in the urine of IC/PBS women measured by thymidine incorporation assay, but no difference between ulcerative and non-ulcerative lesions was found. IL-8 (CXCL-8) can be considered a marker to recognize ulcerative and nonulcerative IC/PBS. Erickson et al. defined, for the first time, a correlation between urinary CXCL-8 and bladder mast cell counts in IC/PBS patients [77] (Table 1).

Moreover, Tyagi et al. reported a high expression of CXCL-8 associated with CXCL-1 and CXCL-10 in the urine of ulcerative women (Table 1). The concentration of cytokines and chemokines is usually higher in spot urine specimens than in plasma samples. The reason may be due to either a lesser dilution of proteins in urine or to lower levels of proteases in urine compared to plasma [6].

**Table 1:** Summary of the main dysregulated proteins described in the text.

Pain Conditions	Site	Gene and Protein Abundances	Type of Abundance	Proteomics Methods
Vulvodynia	vaginal/vestibular areas [35]	Annexin A1, interleukin 1 receptor antagonist, protein S100A9, immunoglobulin G κ chain	up	gel electrophoresis and mass spectrometry
	vaginal fluid [36]	sphingolipid	up	UPLC-MS/MS
Endometriosis	endometrium [42,44]	Genes: *C7*, *CFH*, *FZD7*, *LY96*, *PDLIM3*, *PTGIS*, and *WISP2* Proteins: LY96, PDLIM3, PTGIS, and WISP2 [Bae] MIF (Zhang 2015)	up	microarray
	Plasma [41,45]	proteins related to angiogenesis/cell migration; inflammatory cytokines	up	multiplex aptamer-based proteomics discovery
	Peritoneal fluid [41,46]	proteoforms of 1-antitrypsin and S100-A8, transferrin, 1b-glycoprotein and aptoglobin; inflammatory cytokines	up	2D-PAGE combined with LC-MS/MS
	Endometrial fluid [33]	signal transduction and cytoskeletal structure	up	2D-PAGE)
	eutopic endometrium [52]	P450		hybridization method
	eutopic endometrium [50]	molecular chaperones, proteins involved in protein and DNA formation/breakdown, and secreted proteins	up	2D-PAGE mass spectroscopic
	Serum and eutopic endometrium [54]	proteins of cytoskeletons or involved in the regulation of the cell cycle, signal transduction, or immunological function	up	2D-PAGE
	eutopic endometrium [47]	proteins involved in apoptosis, immune reaction, glycolytic pathway, cell structure, and transcription	up	Immunoblot and immunohistochemical analyses
	eutopic endometrium [49]	structural proteins (vimentin, actins), stress response (Peroxiredoxins, HSP B1, HSP70, HSP90) or signaling (14-3-3 proteins, annexins), protein-folding and protein-turnover, immunity, energy production, signal transduction, RNA biogenesis, protein biosynthesis, and nuclear proteins	up	2D-PAGE Western blot, and MS
	eutopic endometrium [51]	Protein involved in the PI3K/AKT signaling pathway and focal adhesion (the laminin family)	up	LC-MS-MS analysis
		members of the S100 protein family	down	LC-MS-MS analysis
	eutopic endometrium [62]	Humoral immune response pathways, antimicrobial humoral response, and the regulation of the nitric oxide biosynthetic process	up	Tandem mass tags combined with multidimensional liquid chromatography and mass spectrometry analyses
		necroptotic process, regulation of necrotic cell death	down	Tandem mass tags combined with multidimensional liquid chromatography and mass spectrometry analyses
	Follicular fluid [61]	Immunoglobulin heavy constant gamma 2 (IGHG2), glia-derived nexin (GDN), and Inter-alpha-trypsin inhibitor heavy chain H3 (ITIH3)	up	LFQP and PRM
		corticosteroid-binding globulin (CBG), angiotensinogen (AGT), and Fetuin-B (FETUB)	down	LFQP and PRM
Adenomyosis	Adenomyotic tissue [65]	cytoskeleton proteins, HSP and	up	2D_PAGE MALDI-TOF
	serum [66]	immune response, the inflammatory response, and cell adhesion	up.	iTRAQ
	Ectopic endometrium [67]	annexin A2 correlated with markers of epithelial-to-mesenchymal transition enhanced the proangiogenic capacity of adenomyotic endometrial cells through the HIF-1/VEGF-A pathway	up	polyacrylamide gel electrophoresis/MS
	tissue and blood [68]	regulation of cell morphogenesis’ and ‘cytoskeletal organization (HSP90A, STIP1 and TAGLN-2)	up	TEM, LC-MS
Interstitial cystitis/bladder pain syndrome	Bladder biopsy [73]	inflammatory setting (T and B cells markers); endoplasmic reticulum stressed proteins	up	nHPLC-MS/MS
	Bladder biopsy [73]	cellular adhesive proteins, cell proliferation/wound healing Rap1-related proteins	down	nHPLC-MS/MS
		ubiquitination	down	
	NDA tissue [74]	protein; muscarin M2, purinergic P2 × 1, P2 × 2, and histamine receptors	up	multiple antigen bead assay
	bladder submucosa [74]	urothelial markers, focal lymphoid aggregates	down	multiple antigen bead assay
	bladder submucosa [74]	cytokines, chemokines, and enhanced immunoreactivity for muscarinic M2, purinergic P2 × 1, P2 × 2, and histamine H1 receptors	up	multiple antigen bead assay
	Urine samples [76]	tyramine and 2-oxoglutarate phenylacetylglutamin	up	MS
	Urine [77]	CXCL-8 and bladder mast cell counts	up	tryptase stain
		One glycosylated nonapeptide, antiproliferative	-	thymidine incorporation assay

2D-Page: Two-dimensional polyacrylamide gel electrophoresis; LC-MS/MS: Liquid Chromatography—Tandem Mass Spectrometry; MS: mass spectrometry; LFQP: label-free quantitative proteomics; PRM: parallel reaction monitoring; MALDI-TOF: matrix-assisted laser desorption/ionization (MALDI), and the mass analyzer is time-of-flight (TOF); iTRAQ: isobaric tags for relative and absolute quantitation; TEM: transmission electron microscopy; nHPLCMS/MS: nanoscale high-performance Liquid Chromatography–Tandem Mass Spectrometry, UPLC-MS/MS: Ultrahigh-Performance Liquid Chromatography Tandem Mass Spectroscopy.

## 4. Discussion

In this review, we have discussed the most recent results from proteomics studies on women suffering from CPP and VPDs. These studies rely on the idea that different clinical manifestations of chronic pathologies can be related to different proteoforms, with differential biological features and functions. Multiple mechanisms can induce biochemical and biomolecular diversity in cells, such as amino acid variation, alternative RNA splicing, post-translation modification (PTM), and post-translational cleavage by the same gene. The identification and characterization of protein biomarkers are important for diagnosis and personalized treatments [23,24,25,26].

The annexin family has emerged as among the most frequently dysregulated proteins in the gynecological diseases examined in this review. Annexin upregulation has been found in women with vulvodynia (annexin 1) and in adenomyosis samples; interestingly, these proteins are involved in the induction of both metastatic potential and proangiogenic capacity in endometrial cells. Annexin 1 is phospholipid-binding protein active in membrane trafficking and has been related to mast cell granules, one of the most important mechanisms implicated in the genesis of vulvodynia [35]. Annexin 2 is a pleiotropic protein with a mechanism of action fully related to the site of its activity. It mainly connects membrane protein complexes, including ion channels, to the internal actin cytoskeleton and extracellular matrix. Annexin 2 can be considered a marker of epithelial-to-mesenchymal transition, and it is associated with the induction of endometrial cells with both metastatic potential and proangiogenic capacity [63].

Studies on the role of the annexin family were also conducted in oncologic research. Annexin 8 (ANXA8) is involved in cell migration, cell adhesion, and vasculature development, as well as in the regulation of PI3K-Akt and focal adhesion in women with ovarian cancer. Therefore, ANXA8 expression is significantly related to poor prognosis and has been considered a strong candidate as a novel biomarker and therapeutic target for ovarian cancer. As for oncologic pathology, the annexin family plays an important role in adenomyosis, and it might be considered a potential biomarker and therapeutic candidate for adenomyosis, endometriosis, and vulvodynia [78].

PI3K-Akt-mTOR is an important cell signaling pathway, activated by steroid hormones and growth factors that induce cell proliferation and survival [69]. Overactivation of the PI3K-Akt-mTOR pathway has been found in human cancer progression, particularly in gynecological cancers [79].

Studies on the role of this pathway in endometriosis demonstrated that treatment with the tyrosine kinase inhibitor Sunitinib suppressed the migration of ectopic endometrial cells with the involvement of the VEGFR-PI3K-AKT-YBX1-Snail signaling pathway in both in vitro and in vivo experiments. This study suggests that the blockage of tyrosine kinase activity might represent an attractive route for endometriosis therapy [80].

Cytoskeleton proteins, such as actin and vimentin, are upregulated in proteomics samples of endometriosis patients and in eutopic endometrium samples. [81]. Vimentin is an intermediate filament protein involved in the regulation of epithelial–mesenchymal transition (EMT) that affects a diverse range of physiological and pathological processes, such as growth and wound healing [82]. Vimentin is closely related to the occurrence and development of various tumors by modulating the EMT, as well as to oncologic settings; data from literature describe EMT as a physiopathological mechanism implicated in endometriosis cells. Inflammatory mediators involved in retrograde menstrual fluid might contribute to ectopic endometrial EMT and migration in the presence of peritoneal hypoxia. Kusama et al. demonstrated that hypoxic and proinflammatory stimuli induce EMT and cell migration and that inflammation increased the expression of mesenchymal N-cadherin and vimentin in endometrial epithelial cells [83,84].

In the light of these discoveries, cytoskeletal proteins could be considered biomarkers for the early diagnosis of endometriosis and possible therapeutic targets.

Studies performed in interstitial cystitis patients have shown novel therapeutic targets linked to the release of inflammatory cytokines. MCP-1, eotaxin, MIP-1β, TNF-α, and PGE2 were differentially expressed between IC/BPS patients and the control group [85]. Increased mast cells that release proinflammatory cytokines and chemokines in the bladder urothelium were seen in patients with IC/BPS. Tumor necrosis factor-α (TNF-α) is a proinflammatory cytokine involved in bladder damage. Immunotherapies with monoclonal antibody and anti-TNF-α agents, certolizumab pegol and adalimumab, significantly improved clinical symptoms in IC/BPS women through the inhibition of mast cell degranulation [86], highlighting that TNF-α might represent a crucial therapeutic target for IC/BPS. IC/BPS patients with Hunner’s lesions showed a downregulation of proteins involved in ubiquitination compared to those with non-Hunner lesions, as well as a decrease in immune cell adhesion proteins. Similarly, bladder biopsies showed a decreased abundance of proteins involved in peptide metabolism and proteins related to Rap1 signaling, involved in cell proliferation, cell-to-cell adhesion, and cellular junction formation, highlighting the possibility that these pathways might be involved in the pathogenesis of IC/BPS [73]. However, the limited number of patient samples represents an important limitation; therefore, more randomized control trials, with a higher number of recruited women, are necessary to understand the pathophysiology of IC/BPS.

## 5. Conclusions

The above-described discoveries open a new prospective to a better understanding of the pathophysiological processes of CPP and VDP and the development of mechanism-based therapies. Proteomics-based methods represent a crucial approach to identify dysregulated proteins in CPP and VDPs, allowing the identification of new biomarkers that are also useful for an accurate differential diagnosis between distinctive causes of these diseases.

The annexin protein family dysfunction suggests the involvement of the cytoskeleton pathway in the pathogenesis of both adenomyosis and vulvodynia. Similarly, the PI3K-Akt-mTOR signaling pathway, with involvement highlighted in endometriosis samples, as well as the detection of inflammatory cytokines in interstitial cystitis/bladder pain syndrome (IC/BPS), suggests the possibility of common molecular and pathogenic mechanisms between cancer onset and progression and chronic pain disorders.

The PI3K-Akt-mTOR pathway is well-known for its role in cell proliferation and survival and in both tumorigenesis and the pathogenesis of chronic pain. Similarly, inflammatory cytokines are involved in promoting both the tumor environment and pain sensitization in CPP/VDP. Therefore, these findings could represent valuable targets for paving the way for the differential diagnosis and therapeutic management of CPP and VDPs, highlighting the need for a deeper understanding of the overlaps to optimize both the prevention and treatment of these conditions. 

## Data Availability

Not applicable.
